# Systemic inflammatory biomarkers in Schizophrenia are changed by ECT administration and related to the treatment efficacy

**DOI:** 10.1186/s12888-023-05469-2

**Published:** 2024-01-17

**Authors:** Yu Wang, Guangfa Wang, Muxin Gong, Yujing Yang, Yuru Ling, Xinyu Fang, Tingting Zhu, Zixu Wang, Xiangrong Zhang, Caiyi Zhang

**Affiliations:** 1 Department of Geriatric Psychiatry, The Affiliated Brain Hospital of Nanjing Medical University, 210029 Nanjing, Jiangsu China; 2 The Affiliated Xuzhou Oriental Hospital of Xuzhou Medical University, 221004 Xuzhou, Jiangsu China

**Keywords:** Schizophrenia, Electroconvulsive therapy, Inflammation, Neutrophils, Lymphocytes, NLR

## Abstract

Immune inflammation has long been implicated in the pathogenesis of schizophrenia. Despite as a rapid and effective physical therapy, the role of immune inflammation in electroconvulsive therapy (ECT) for schizophrenia remains elusive. The neutrophils to lymphocytes (NLR), platelets to monocytes (PLR) and monocytes to lymphocytes (MLR) are inexpensive and accessible biomarkers of systemic inflammation. In this study, 70 schizophrenia patients and 70 age- and sex-matched healthy controls were recruited. The systemic inflammatory biomarkers were measured before and after ECT. Our results indicated schizophrenia had significantly higher peripheral NLR, PLR and MLR compared to health controls at baseline, while lymphocytes did not differ. After 6 ECT, the psychiatric symptoms were significantly improved, as demonstrated by the Positive and Negative Syndrome Scale (PANSS). However, there was a decline in cognitive function scores, as indicated by the Mini-Mental State Examination (MMSE). Notably, the neutrophils and NLR were significantly reduced following ECT. Although lymphocytes remained unchanged following ECT, responders had significantly higher lymphocytes compared to non-responders. Moreover, the linear regression analyses revealed that higher lymphocytes served as a predictor of larger improvement in positive symptom following ECT. Overall, our findings further highlighted the presence of systemic inflammation in schizophrenia patients, and that ECT may exert a therapeutic effect in part by attenuating systemic inflammation. Further research may therefore lead to new treatment strategies for schizophrenia targeting the immune system.

**Supplementary Information:**

The online version contains supplementary material available at 10.1186/s12888-023-05469-2.

## Introduction

Schizophrenia is a devastating psychiatric disorder that has a detrimental impact on a considerable population worldwide, severely limiting psychosocial functioning and imposing a significant burden on society [1]. Epidemiological studies have frequently linked maternal infections to the development of schizophrenia in offspring [2]. Moreover, environmental factors such as autoimmune diseases, and acute stress could increase maternal immune responses, leading to an elevated risk of schizophrenia [3]. Notably, growing evidence suggests that dysregulation in innate and adaptive branches of the immune system plays a role in schizophrenia [4].

Neutrophils are the first line of defense against infection and can induce inflammation and oxidative stress. A recent study reported that neutrophils were positively correlated with the severity of psychiatric symptoms and the reduced brain grey matter volume in first-episode schizophrenia [5]. However, lymphocytes play an important role in adaptive immunity, with both regulatory and protective functions and low levels reflecting poor health conditions [6]. Since the blood-brain barrier (BBB) may be impaired under certain pathological conditions, especially in schizophrenia [7, 8], allowing for the entry of peripheral cells into the brain [9]. Actually, evidence has suggested neutrophils may exert a destructive effect on the cerebral tissue after infiltrating the brain [10, 11]. Notably, the infiltration of monocytes into the brain may lead to neuronal damage in mice [12]. Conversely, most studies showed that lymphocytes appear to have a protective effect on the brain [10], and the lack of lymphocytes led to an aggravation of symptoms in animal models of Alzheimer’s disease [13]. The neutrophil-to-lymphocyte ratio (NLR) is the ratio of neutrophils active in the innate immune response to lymphocytes active in the adaptive immune response [14, 15], which is a well-established marker of immune system homeostasis. NLR was originally developed to reflect systemic inflammation in critically ill patients, and was reported to have prognostic value in a range of diseases, including diabetes [16], cancer [17], etc. The platelet-to-lymphocyte ratio (PLR) is an indicator derived from platelet aggregation and systemic inflammation [18], while the monocyte-to-lymphocyte ratio (MLR) serves as a potential peripheral marker of microglia activation in the brain, as monocytes could be recruited to the brain under inflammatory conditions where they interact with or differentiate into microglia [19]. Collectively, the three ratios are important biomarkers of systemic inflammation that can be calculated directly from a complete blood count test, which has been demonstrated to play a role in various neuropsychiatric disorders, including depression, autism and other diseases [20–23]. Most importantly, recent research indicated schizophrenia patients had significantly higher NLR, PLR and MLR compared to healthy controls [24–27].

Electroconvulsive therapy (ECT) is a fast-acting physical therapy for schizophrenia [28]. Evidence has suggested ECT combined with clozapine was more effective than antipsychotics [29]. Moreover, a recent study indicated schizophrenia patients treated with ECT showed lower readmissions [30]. Although studies suggested that ECT may cause cognitive impairment, recent research showed that the effects of ECT on cognitive function are mildly tolerable, transient and reversible [31, 32]. Notably, some studies even showed improved results [31, 32]. Despite being a fast and effective form of physical therapy for schizophrenia [33], the mechanisms of ECT are rarely explored. Given the possible immunomodulatory effects of ECT [34], observing the cost-effective and readily available systemic inflammatory biomarkers following ECT can help explore the role of inflammation in ECT for schizophrenia. In the present study, we compared the systemic inflammatory biomarkers between schizophrenia patients and healthy controls, and aimed to investigate changes in systemic inflammatory biomarkers following ECT and its relationship with clinical efficacy.

## Materials and methods

### Participants

Schizophrenia patients were recruited from Xuzhou Oriental People’s Hospital. Inclusion criteria were as follows: (1) age between18-65 years; (2) diagnosis of schizophrenia according to ICD-10 diagnostic criteria; (3) Indication for ECT under the assessment of experienced clinicians and anesthesiologists, including schizophrenia of intense psychomotor agitation or retardation, suicide attempts, pronounced aggressive behavior, intolerance to pharmacotherapy, and lack of response to previous antipsychotic treatment at adequate doses and durations. The exclusion criteria were (1) history of ECT within 6 months; (2) brain disease such as Alzheimer’s Disease, Parkinson’s Disease, stroke et al.; immune disease such as SLE (systemic Lupus Erythematosus), asthma, systemic Sclerosis et al.; (3) recent (three months) infection, fever, or using anti-inflammatory drugs; (4) lactating and premenopausal women; and (5) use of alcohol, narcotics and other psychoactive substances. The healthy controls were recruited from the physical examination center of Xuzhou Medical University Affiliated Hospital. They were matched with patients for age and sex and had no history of psychiatric disorders or inflammatory events recently (three months). All participants signed a written informed consent form, and the ethical approval was obtained Ethics Committee of Xuzhou Oriental People’s Hospital. During ECT, 5 patients dropped out from the study (3 patients were excluded due to fever and 2 patients were excluded due to controversial diagnosis). Therefore, a total of 140 participants (70 patients and 70 matched healthy controls) were included in the follow-up analyses.

### ECT procedure

Patients received bilateral temporal ECT combined with antipsychotic medication (all patients had been on antipsychotic medication for a fortnight prior to ECT). ECT (Thymatron System IV Integrated ECT Instrument) was administered twice or three times a week. Methohexital (0.75 mg/kg-1.0 mg/kg) and succinylcholine (0.5 mg/kg–1.0 mg/kg) were used for anesthesia and muscle relaxation, respectively. During ECT, the type and dose of antipsychotic drugs kept constant and have been converted to chlorpromazine equivalents. The study focused on observing patients at two time points: pre-ECT, and after six ECT sessions (post-ECT). However, the study did not affect the treatment plan. Patients who did not meet the criteria for recovery after six ECT sessions continued to receive ECT and medication based on their individual conditions. During ECT, seven patients were treated with antidepressants for mild depressed mood (sertraline n = 3, 50 mg/day; paroxetine, n = 4, 20 mg/day); two patients with lithium carbonate (0. 3 g/day) for unstable mood; five patients with propranolol (10 mg/day) for rapid heart rate; three patients with benzodiazepines for anxiety (lorazepam n = 1, 1.5 mg/day; clonazepam n = 1, 2 mg/day; alprazolam n = 1, 0.2 mg/day), and 14 patients used non-benzodiazepine aids for sleep problems (zopiclone n = 14, 7.5 mg/day).

### Clinical assessments

The PANSS was used to assess the severity of psychiatric symptoms during ECT. The reduction rate in the PANSS scores was calculated by the formula: (*pre-PANSS– post-PANSS*) / *(pre-PANSS − 30)* × 100%, where *pre-PANSS* is the score at baseline and *post-PANSS* is the score at follow-up. Patients were defined as responders when the reduction rate of the PANSS total score was more than 50% [35]. Moreover, the Mini-Mental State Examination (MMSE) was used to assess cognitive function during ECT.

### Blood cell analysis

Fasting blood samples were collected 06:00–07:00 before the first ECT and 1 day following 6 ECT, and fasting control blood samples were taken 07:30–09:30 on assessment days. The samples were collected in an EDTA anticoagulation tube, and performed using the Sysmex XN-1000 fully automated hematology analyzer developed by Sysmex Japan. The test results included PLT (10^9/L), neutrophils (10^9/L), lymphocytes (10^9/L), monocytes (10^9/L), and three important ratios PLR, NLR and MLR.

### Statistical analysis

Demographic and clinical data were managed and analyzed using IBM SPSS Statistics v.25 and GraphPad prism 9.0.0. Data was presented as mean (standard deviation, SD) or number (%) per group where appropriate. Chi-square tests were used to calculate group differences regarding smoking status and gender. Data were tested for normality using the Shapiro-Wilk or Kolmogorov-Smirnov test and Q-Q plots, which has shown most data were not normally distributed. Thus, logarithmic transformation was used to transform the blood test values (including PLT, neutrophils, lymphocytes, monocytes, and three important ratios PLR, NLR and MLR) to achieve normal distribution. Independent Samples t-test was used to compare differences in systemic inflammatory biomarkers between healthy controls and schizophrenia, and Paired Samples t-test was used to identify changes in systemic inflammatory biomarkers following ECT. Linear correlation analysis was employed to explore the associations between systemic inflammatory biomarkers and psychiatric symptoms, with age, gender, BMI, education, duration of illness, and chlorpromazine equivalent doses included as covariates. Response to ECT in terms of psychiatric symptom, positive symptom, negative symptom and cognitive function was measured as the variation of PANSS, P-PANSS, N-PANSS and MMSE from pre-ECT to post-ECT that we indicated as Δ PANSS, Δ P-PANSS, Δ N-PANSS and Δ MMSE. All statistical tests were two-tailed, with *P* < 0.05 being significant.

## Results

### Schizophrenia patients showed elevated systemic inflammatory biomarkers compared to health controls

Demographic and clinical characteristics were listed in Table 1. A total of 140 participants (70 schizophrenia patients and 70 health controls) were included in the study. The results showed significant differences in BMI and education (t=-2.01, *P* = 0.044; t=-3.16, *P* = 0.002, respectively), while no differences in age, gender distribution and smoking between the two group (all *P* > 0.05) (Table 1).

**Table 1:** Demographic and clinical characteristics between schizophrenia patients and healthy controls

	HCs(n = 70)	SCZ(n = 70)	t/c2	*P* value	95% CI of the Difference	
					Lower	upper
Age	35.80(9.90)	35.54(10.35)	t=-0.12	0.905	-3.00	3.00
Male gender, n (%)	39(55.7%)	39(55.7%)	c2 = 0	1.000		
BMI	23.58(3.63)	24.69(4.06)	t=-2.01	**0.044**	0.03	2.61
Education (years)	13.33(2.67)	11.40(3.40)	t=-3.16	**0.002**	-3.00	0.00
Smokers, n (%)	26(37.1%)	20(28.6%)	c2 = 1.17	0.280	0.48	1.24
Family history, n (%)		13(18.6%)			0.09	0.28
Duration of illness (months)		124.77(111.28)			98.24	151.31
PLT (10^9/L)	2.36(0.07)	2.41(0.12)	t=-2.781	**0.006**	-0.08	-0.01
Neutrophils (10^9/L)	0.61(0.08)	0.74(0.14)	t=-6.639	**< 0.0001**	-0.17	-0.09
Lymphocytes (10^9/L)	0.46(0.06)	0.46(0.09)	t = 0.131	0.896	-0.02	0.03
Monocytes (10^9/L)	0.12(0.03)	0.19(0.05)	t=-7.613	**< 0.0001**	-0.07	-0.04
NLR	0.42(0.07)	0.54(0.15)	t=-6.028	**< 0.0001**	-0.16	-0.08
PLR	2.08(0.09)	2.14(0.16)	t=-2.434	**0.016**	-0.10	-0.01
MLR	0.07(0.02)	0.11(0.04)	t=-7.264	**< 0.0001**	-0.05	-0.03
Chlorpromazine equivalent doses		506.29(242.31)			448.51	564.06
Antidepressants, n (%)		7(10.0%)			0.03	0.17
Lithium, n (%)		2(2.9%)			-0.01	0.07
Propranolol, n (%)		5(7.1%)			0.01	0.13
Benzodiazepines, n (%)		3(4.3%)			-0.01	0.09
Non-benzodiazepine hypnotics, n (%)		14(20.0%)			0.10	0.30

Abbreviations: HCs, health controls; SCZ, schizophrenia; CI, Confidence Interval; BMI, Body Mass Index; PLT, Platelet; NLR, Neutrophils to Lymphocytes; PLR, PLT to Lymphocytes; MLR, Monocytes to Lymphocytes. Values are bolded when the *p*-value <= 0.05.

**Fig. 1:**
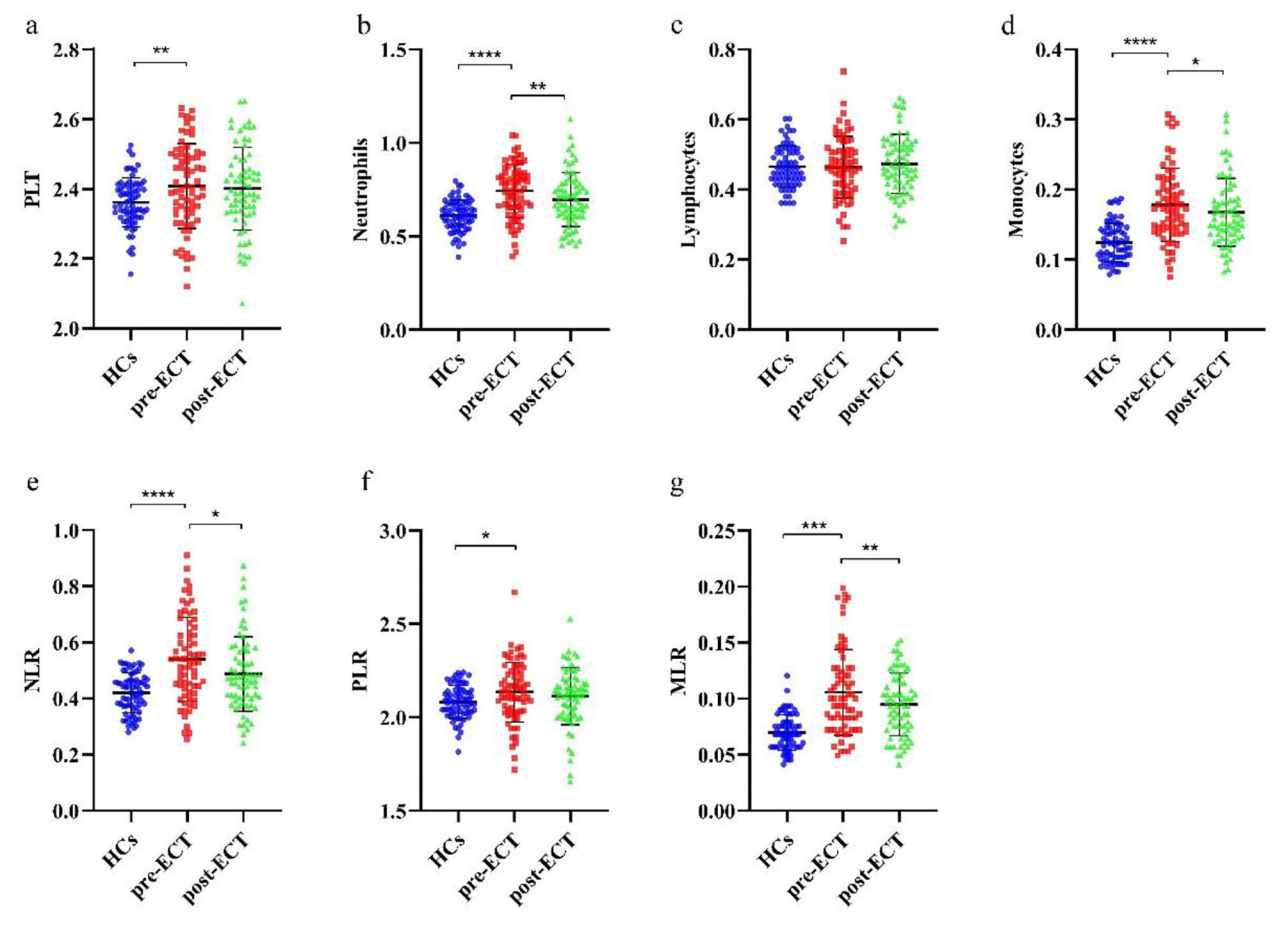
Differences in systemic inflammatory biomarkers in schizophrenia and controls and changes following ECT. Data was expressed as mean ± standard deviation (SD). a PLT was significantly elevated in schizophrenia compared to healthy controls (HCs) but remained stable following ECT. b Neutrophils was significantly elevated in schizophrenia and reduced following ECT. c Lymphocytes showed no differences between schizophrenia and HCs and kept stable following ECT. d Monocytes were significantly elevated in schizophrenia and decreased following ECT. e the neutrophil-to-lymphocyte ratio (NLR) was significantly higher in schizophrenia and reduced following ECT. f the PLT-to-lymphocyte ratio (PLR) was elevated in schizophrenia, while demonstrated no differences following ECT. g the monocytes- to-lymphocyte ratio (MLR) was higher in schizophrenia and significantly downregulated following ECT. *p < 0.05, **p < 0.01, ***p < 0.001, **** *p* < 0.0001

At baseline, schizophrenia showed significantly higher PLT (t=-2.781, *P* = 0.006), neutrophils (t=-6.639, *P* < 0.0001), monocytes (t=-7.613, *P* < 0.0001), NLR (t=-6.028, *P* < 0.0001), PLR (t=-2.434, *P* = 0.016) and MLR (t=-7.264, *P* < 0.0001) compared to health controls, while lymphocytes (t = 0.131, *P* = 0.896) did not differ (Fig. 1).

### The elevated systemic inflammatory biomarkers were reduced following ECT in Schizophrenia patients

After ECT, the PANSS scores and its subscales (P-PANSS and N-PANSS) were significantly decreased, with a 61.76% reduction in total PANSS scores. In addition, the MMSE scores were significantly reduced following ECT.

Considering the systemic inflammatory biomarkers, the results indicated the neutrophils (t=-2.684, *P* = 0.009), monocytes (t=-2.102, *P* = 0.039), NLR (t=-2.57, *P* = 0.012) and MLR (t=-2.725, *P* = 0.008) were significantly reduced following 6 ECT sessions. However, PLT (t=-0.768, *P* = 0.445), lymphocytes (t = 1.066, *P* = 0.29) and PLR (t=-1.353, *P* = 0.181) remained unchanged following ECT (Supplementary Tables 1 and Fig. 1).

### Higher lymphocytes levels were associated with better improvement in psychiatric symptoms following ECT

We subsequently used the reduction rate of PANSS to assess treatment responses to ECT, and patients with a PANSS reduction rate of ≥ 50% was defined as responders [35]. Overall, 52 of 70 schizophrenia patients were responders, while 18 patients were non-responders. Moreover, there were no differences in age, BMI, education, duration of illness and chlorpromazine equivalent doses between responders and non- responders (all *P* > 0.05).

Regarding systemic inflammation biomarkers, the results showed responders had significantly higher baseline lymphocytes (t=-2.808, *P* = 0.006) compared to non-responders, while other showed no differences between the two groups (Supplementary Table 2).

Since responders exhibited higher lymphocytes compared to non-responders. Subsequently, we applied linear regression to explore the association of lymphocytes and the improvement of psychiatric symptoms. The results indicated a significant association between lymphocytes and ΔP-PANSS (F = 2.797, *p* = 0.014), while lymphocytes showed no correlation with other scales (all *p* > 0.05). Notably, higher baseline lymphocytes levels were identified as a significant predictor of the decrease inΔP-PANSS following ECT (β = 23.032, *p* = 0.007). In addition, the results showed that chlorpromazine equivalent doses were negatively associated with ΔP-PANSS (β = -0.007, *p* = 0.030) (Table 2).

**Table 2:** Higher baseline lymphocytes were associated with larger improvement in ΔP-PANSS following ECT

Variables	β	*P* value	95% CI for β	
			Lower	upper
age	0.043	0.608	-0.125	0.211
gender	-0.514	0.734	-3.527	2.499
BMI	0.238	0.209	-0.137	0.613
Education(years)	-0.346	0.117	-0.780	0.089
Duration of illness(months)	-0.005	0.486	-0.020	0.009
Chlorpromazine equivalent doses	-0.007	**0.030**	-0.012	-0.001
Lymphocytes (10^9/L)	23.032	**0.007**	6.676	39.388

Abbreviations: CI, Confidence Interval; Δ P-PANSS, Response to ECT in terms of positive symptom was measured as the variation of P-PANSS from pre-ECT to post-ECT. Values are bolded when the *p*-value <= 0.05

## Discussions

To our knowledge, this is the first and most comprehensive report to date on the effects of ECT on systemic inflammatory biomarkers in schizophrenia. Schizophrenia patients showed significantly higher PLT, neutrophils, monocytes, and the three important ratios NLR, PLR and MLR compared to healthy controls. Notably, neutrophils, NLR were significantly reduced following ECT. Moreover, responders exhibited higher lymphocytes compared to non-responders, and the linear regression analyses reveled that higher lymphocyte levels were a predictor of better improvement of positive psychiatric symptom following ECT.

At baseline, schizophrenia patients showed significantly higher PLT, neutrophils, monocytes, NLR, PLR and MLR compared to healthy controls, replicating previously findings [36, 37]. The relationship between systemic inflammatory biomarkers and psychiatric symptoms remains elusive. While some studies reported a positive correlation between NLR and total PANSS scores, others reported NLR did not correlate with clinical symptoms [38, 39]. After 6 ECT, the neutrophils, monocytes, NLR and MLR were significantly reduced in patients with schizophrenia. Together with a significant reduction in pro-inflammatory factor TNF-α after 10 sessions of ECT suggests that ECT may work in part by reducing inflammation in the treatment of schizophrenia [40]. Few studies have examined the relationship between systemic inflammatory biomarkers and response to ECT in schizophrenia. Previous study found anti-inflammatory factor IL-4 was elevated after 9 ECT, and IL-4 was negatively correlated with BPRS, while NF-κB, a major pathway involved in the immune inflammation response, did not change following ECT [41]. However, previous studies suggested BBB may be disrupted in schizophrenia, thus allowing more circulating cytokines as well as leukocytes to infiltrate the brain [42]. In this case, the elevated peripheral inflammatory signals may be transmitted to the brain, these signals can activate microglia, the brain’s resident macrophages, ultimately leading to alterations in neuroplasticity and neurotransmitters [43], which is consistent with the elevated microglia activity observed in schizophrenia [44]. Notably, previous animal studies using ECS to mimic the function of ECT found that ECS had the ability to reduce the activity of microglia in both normal animal models [45, 46] and schizophrenia animal models [47]. The findings thus suggest ECT may treat schizophrenia in part by reducing inflammation.

Although previous studies suggested lymphocytes were significantly lower in schizophrenia patients [48, 49], lymphocytes did not differ in our study, which may be due to the fact that our patients had received at least 2 weeks of antipsychotic medication prior to inclusion in the study [37]. Furthermore, although lymphocytes did not change after ECT in our study, the results indicated responders had significantly higher lymphocytes compared to non-responders. Moreover, although not schizophrenia animal models, a recent study showed the expression of several immune checkpoint genes, particularly lymphocyte-activating gene-3 (Lag3), was the only microglial transcript significantly reduced by electroconvulsive stimulation (ECS) [50]. Notably, previous study suggested a negative correlation between lymphocytes and PANSS [48], our findings indicated lymphocytes served as a predictor for greater improvement in positive symptom following ECT, which provided further evidence suggesting lymphocytes may be a protective factor for schizophrenia. Actually, a study showed active enhancer enrichment in CD19 and CD20 lymphocytes in 108 significant loci in schizophrenia [51]. The subsets of lymphocytes such as regulatory T cells (Tregs) and B cells (Bregs) could prevent chronic inflammation by suppressing immune responses, down-regulating production of leukocytes and pro-inflammatory cytokines, which may influence the development of schizophrenia though affecting brain development, immunity, etc [52]. Furthermore, previous studies showed schizophrenia had disrupted lymphocyte subsets [53], and a meta-analysis demonstrated first-episode schizophrenia had elevated CD4/CD8 and significantly lower CD3% [54]. Additionally, CD3 levels were significantly elevated following antipsychotic treatment, and the reduction in CD3 was consistent with reduced levels of the anti-inflammatory factor IL-2. In contrast, CD19 B lymphocytes were elevated in schizophrenia and reduced after treatment [55], suggesting different lymphocyte subsets may have different roles in schizophrenia, highlighting the importance to explore different lymphocyte subsets following ECT in schizophrenia patients.

The increased peripheral blood cytokines were associated with cognitive deficits and reduced brain volume in schizophrenia [56]. In this study, the MMSE scores were reduced after ECT. Actually, the activation of monocytes has been consistently reported in schizophrenia, and first-episode schizophrenia had higher MCP-1 (Monocyte chemoattractant protein-1) both in the cerebrospinal fluid and blood [57]. Moreover, a recent study demonstrated atypical monocyte genes were negative correlated with cortical thickness and cognitive function in healthy controls, however, the correlation was attenuated in schizophrenia [58], highlighting the importance to explore monocyte subsets in the deterioration of cognitive function after ECT. Moreover, since peripheral systemic inflammation can lead to microglia activation in the brain, in vivo assessment of microglia activity before and after ECT by 18-kDa translocator protein (TSPO) PET can help to further clarify whether ECT treats schizophrenia by reducing neuroinflammation [59, 60].

Several limitations of this current study should be mentioned here. Firstly, although the chlorpromazine equivalent has a minimal impact on the improvement of psychiatric symptom following ECT in our study (β=-0.007, *p* = 0.030), and the type and doses of antipsychotics kept stable during ECT, antipsychotics may affect systemic inflammatory biomarkers [25]. Thus, including a group of schizophrenia patients who only take antipsychotics could help determine whether the reduction in inflammation is due to ECT or simply a result of other treatment and environmental factors surrounding ECT. Secondly, a follow-up study is needed to further determine whether changes in systemic inflammatory biomarkers were due to disease status or the effects of ECT. Thirdly, we used MMSE to assess the cognitive function of schizophrenia, which is not as comprehensive as MCCB (MATRICS Consensus Cognitive Battery). Ultimately, future research considering the drug interference, combining clinical and animal studies, and utilizing more appropriate cognitive assessment tools, may provide a more adequate understanding of the role of inflammation in ECT for schizophrenia.

## Conclusions

In conclusion, our findings further highlighted the role of inflammation in the pathogenesis of schizophrenia, as demonstrated by the elevated NLR in schizophrenia patients. Notably, NLR was significantly reduced following ECT, indicating that ECT may treat schizophrenia by reducing inflammation. Furthermore, lymphocytes may play a protective role, as they were significantly higher in responders, and served as a significant predictor for greater positive symptom improvement following ECT.

### Electronic supplementary material

Below is the link to the electronic supplementary material.

Supplementary Material 1

## Data Availability

All data generated or analyzed during this study were included in this published article.
