# Supplementary material for: Systemic inflammatory biomarkers in Schizophrenia are changed by ECT administration and related to the treatment efficacy
Source: BMC Psychiatry. 2024 Jan 17;24:53. doi: 10.1186/s12888-023-05469-2 (PMC10792810; doi:10.1186/s12888-023-05469-2)
Supplement: Supplementary file 1 — Supplementary Material 1 [file 12888_2023_5469_MOESM1_ESM.docx]

**Title Page**

**Systemic inflammatory biomarkers in Schizophrenia are Changed by ECT Administration and related to the treatment efficacy**

**Running title:** Systemic inflammatory biomarkers following electroconvulsive therapy in schizophrenia.

Yu Wang^1, #^, Guangfa Wang^2, #^, Muxin Gong^2, #^, Yujing Yang^2^, Yuru Ling^1^, Xinyu Fang^1^, Tingting Zhu^1^, Zixu Wang^1^, Xiangrong Zhang ^1, 2, *^, Caiyi Zhang^2, **^

^1^ Department of Geriatric Psychiatry, The Affiliated Brain Hospital of Nanjing Medical University, Nanjing, Jiangsu, China.

^2^ The Affiliated Xuzhou Oriental Hospital of Xuzhou Medical University, Xuzhou, Jiangsu, 221004, China

^#^ These authors contributed to this work equally. They should be regarded as Joint First Author.

**Corresponding author:**

* Corresponding author: Department of Geriatric Psychiatry, The Affiliated Brain Hospital of Nanjing Medical University, Nanjing, Jiangsu, 210029, China

** Corresponding author: Department of Geriatric Psychiatry, The Affiliated Xuzhou Oriental Hospital of Xuzhou Medical University, Xuzhou, Jiangsu, 221004, China

E-mail address: drxrz@hotmail.com (X. Zhang);

100002018003@xzhmu.edn.cn (C. Zhang).

**Table S1.** Changes of systemic inflammatory biomarkers following ECT in schizophrenia.

|  | pre-ECT | post-ECT | t | P | 95% CI of the Difference | |
| --- | --- | --- | --- | --- | --- | --- |
|  |  |  |  |  | Lower | upper |
| PLT (10^9/L) | 2.41(0.12) | 2.40(0.12) | t=-0.768 | 0.445 | -0.026 | 0.011 |
| Neutrophils (10^9/L) | 0.74(0.14) | 0.70(0.14) | t=-2.684 | **0.009** | -0.082 | -0.012 |
| Lymphocytes (10^9/L) | 0.46(0.09) | 0.47(0.08) | t=1.066 | 0.29 | -0.008 | 0.026 |
| Monocytes (10^9/L) | 0.19(0.05) | 0.17(0.05) | t=-2.102 | **0.039** | -0.020 | -0.001 |
| NLR | 0.54(0.15) | 0.49(0.13) | t=-2.57 | **0.012** | -0.092 | -0.012 |
| PLR | 2.14(0.16) | 2.11(0.15) | t=-1.353 | 0.181 | -0.054 | 0.010 |
| MLR | 0.11(0.04) | 0.09(0.03) | t=-2.725 | **0.008** | -0.019 | -0.003 |
| P-PANSS | 26.19(5.09) | 11.43(4.64) | t=-7.26 | **<0.0001** | -16.50 | -13.50 |
| N-PANSS | 24.13(5.93) | 15.29(4.68) | t=-6.90 | **<0.0001** | -10.00 | -7.00 |
| PANSS | 99.13(12.77) | 56.13(13.91) | t=-7.27 | **<0.0001** | -47.00 | -38.50 |
| MMSE | 25.33(4.32) | 23.69(4.94) | t=-2.75 | **0.0060** | 0.50 | 2.50 |
| Reduction rate of PANSS | 61.76% | |  |  | 56.93 | 66.58 |

**Abbreviations:** CI, Confidence Interval; PLT, Platelet; NLR, Neutrophils to Lymphocytes; PLR, PLT to Lymphocytes; MLR, Monocytes to Lymphocytes; P-PANSS, Positive symptom scores of the PANSS scale; N-PANSS, Negative symptom scores of the PANSS scale; PANSS, (Positive and negative symptom scale); MMSE, Mini-Mental State Examination; Reduction rate of the PANSS, (*pre-PANSS – post-PANSS*) / *(pre-PANSS - 30)* × 100%; Values are bolded when the p-value <= 0.05.

**Table S2.** Systemic inflammatory biomarkers in remitters versus non-remitters

|  | Responders  (n=52) | non-Responders (n=18) | t | P | 95% CI of the Difference | |
| --- | --- | --- | --- | --- | --- | --- |
|  |  |  |  |  | Lower | upper |
| Age | 35.25(10.61) | 36.39(9.82) | t=0.40 | 0.6906 | -4.55 | 6.82 |
| Male gender, n (%) | 28(53.8%) | 11(61.1%) | t=0.29 | 0.5930 | 0.73 | 1.77 |
| BMI | 24.80(4.51) | 24.39(2.44) | t=-0.36 | 0.7165 | -2.64 | 1.82 |
| Education(years) | 11.31(3.34) | 11.67(3.65) | t=0.38 | 0.7023 | -1.51 | 2.22 |
| Duration of illness(months) | 123.08(112.58) | 129.67(110.50) | t=0.22 | 0.8304 | -54.56 | 67.74 |
| Chlorpromazine equivalent doses | 500.38(249.32) | 523.33(226.74) | t=0.34 | 0.7318 | -110.13 | 156.03 |
| PLT (10^9/L) | 2.41(0.12) | 2.39(0.14) | t=-0.636 | 0.527 | -0.088 | 0.045 |
| Neutrophils (10^9/L) | 0.76(0.15) | 0.70(0.14) | t=-1.388 | 0.17 | -0.132 | 0.024 |
| Lymphocytes (10^9/L) | 0.48(0.09) | 0.42(0.08) | t=-2.808 | **0.006** | -0.110 | -0.019 |
| Monocytes (10^9/L) | 0.18(0.05) | 0.16(0.04) | t=-1.513 | 0.135 | -0.050 | 0.007 |
| NLR | 0.53(0.16) | 0.55(0.13) | t=0.489 | 0.626 | -0.062 | 0.102 |
| PLR | 2.11(0.55) | 2.19(0.18) | t=1.846 | 0.069 | -0.006 | 0.166 |
| MLR | 0.10(0.04) | 0.11(0.03) | t=0.598 | 0.552 | -0.015 | 0.027 |

**Abbreviations:** Patients were defined as responders when the reduction rate of the PANSS total score was more than 50%; CI, Confidence Interval; PLT, Platelet; NLR, Neutrophils to Lymphocytes; PLR, PLT to Lymphocytes; MLR, Monocytes to Lymphocytes; Values are bolded when the p-value <= 0.05.
